# The potential regulatory mechanisms of the gonadotropin-releasing hormone in gonadotropin transcriptions identified with bioinformatics analyses

**DOI:** 10.1186/s12958-017-0264-3

**Published:** 2017-06-17

**Authors:** Wei Xiang, Baoyun Zhang, Fenglin Lv, Guangde Feng, Long Chen, Fang Yang, Ke Zhang, Chunyu Cao, Pingqing Wang, Mingxing Chu

**Affiliations:** 1 College of Bioengineering, Chongqing University, Chongqing, 400030 China; 2 Sichuan TQLS Animal Husbandry Science and Technology Co.,LTD, City, Mianyang, Sichuan 621000 China; 3 Key Laboratory of Farm Animal Genetic Resources and Germplasm Innovation of Ministry of Agriculture, Institute of Animal Science, Chinese Academy of Agricultural Sciences, Beijing, 100193 China

**Keywords:** Molecular mechanisms, GnRH signalling pathway, Bioinformatics analyses, Metabolic status

## Abstract

**Background:**

The regulation of gonadotropin synthesis and release by gonadotropin­releasing hormone (GnRH) plays an essential role in the neuroendocrine control of reproduction. However, the mechanisms underlying gonadotropin regulation by GnRH pulse frequency and amplitude are still ambiguous. This study aimed to explore the molecular mechanisms and biological pathways associated with gonadotropin synthesis by GnRH pulse frequencies and amplitudes.

**Methods:**

Using GSE63251 datasets downloaded from the Gene Expression Omnibus (GEO), differentially expressed genes (DEGs) were screened by comparing the RNA expression from the GnRH pulse group, the GnRH tonic group and the control group. Pathway enrichment analyses of DEGs was performed, followed by protein-protein interaction (PPI) network construction. Furthermore, sub-network modules were constructed by ClusterONE and GO function and pathways analysed by DAVID. In addition, the relationship between the metabolic pathways and the GnRH pathway was verified in vitro.

**Results:**

In total, 531 common DEGs were identified in GnRH groups, including 290 up-regulated and 241 down-regulated genes. DEGs predominantly enriched in 16 Kyoto Encyclopedia of Genes and Genomes (KEGG) pathways, including 11 up-regulated pathways (signallingsignallingmetabolic pathways, signallingand GnRH signalling pathway) and 5 down-regulated pathways (type II diabetes mellitus). Moreover, FBJ osteosarcoma oncogene (FOS) and jun proto-oncogene (JUN) had higher connectivity degrees in the PPI network. Three modules in the PPI were identified with ClusterONE. The genes in module 1 were significantly enriched in five pathways, including signallingthe insulin resistance and GnRH signalling pathway. The genes in modules 2 and 3 were mainly enriched in metabolic pathways and steroid hormone biosynthesis, respectively. Finally, knockdown leptin receptor (LEPR) and insulin receptor (INSR) reversed the GnRH-modulated metabolic related-gene expression.

**Conclusions:**

The present study revealed the involvement of GnRH in the regulation of gonadotropin biosynthesis and metabolism in the maintenance of reproduction, achieved by bioinformatics analyses. This, indicates that the GnRH signalling pathway played a central linkings role in reproductive function and metabolic balance. In addition, the present study identified the difference response between GnRH pulse and GnRH tone, indicated that abnormal GnRH pulse and amplitude may cause disease, which may provide an improved understanding of the GnRH pathway and a new insight for disease diagnosis and treatment.

## Background

The hypothalamic-pituitary-gonadal (HPG) axis governs almost all mammalian reproduction events, from foetal development, through puberty to sexual maturity [1]. Regulation of the reproductive system is initiated by an array of external and internal inputs, such as photoperiod, metabolic products and nutrients, growth factors, stress, infection and inflammation, as well as many central and peripheral growth factors and hormones. These inputs are integrated in the brain and hypothalamus to regulate the biosynthesis and secretion of gonadotropin­releasing hormone GnRH [2]. GnRH, a hypothalamic decapeptide, is well known to be4 a crucial factor in normal gonadal development and function [3, 4]. Starting at the onset of puberty, GnRH is released in pulses from axon terminals at the median eminence into the hypothalamic-hypophyseal portal system and then acts on gonadotrophins in the anterior pituitary gland, which express the GnRH receptor. Upon receptor binding, GnRH signalling regulates both the production and release of gonadotrophins, luteinizing hormone (LH) and follicle-stimulating hormone (FSH), which in turn act on their respective receptors in the gonads to stimulate steroid hormone release into the bloodstream [5–7]. LH and FSH then act on the ovary and testis to stimulate the production of gametes, and steroid and peptide hormones [8]. These hormones positively and negatively feedback at the hypothalamus and pituitary level, thus regulating the reproductive hormone cascade [8]. Therefore, understanding the molecular mechanisms involved in the response of gonadotropins to GnRH may help discover new therapeutic targets for reproductive disorders and hormone-dependent malignancies.

GnRH is released intermittently pulses, and changes in GnRH pulse frequencies and amplitudes have differential effects on FSH and LH synthesis and release [9–11]. High GnRH pulse frequencies preferentially stimulate LH synthesis and secretion, whereas FSH synthesis and secretion are preferentially stimulated at lower frequencies. Studies using rodent models have demonstrated that FSHβ gene expression is optimally stimulated by GnRH pulses every 120 min, whereas LHβ gene expression is higher at shorter pulse intervals, every 30 min [10, 12]. The pulsatile manner by which GnRH is released serves as a primary mechanism by which the synthesis and secretion of FSH and LH from the gonadotropes are controlled. Changes in the pulse frequency and amplitude of GnRH release result in differential FSH and LH synthesis and secretion, contributing to regulating the female menstrual cycle [9, 11]. However, the mechanistic basis for the ability of gonadotropes to distinguish pulse frequency and amplitude are still ambiguous, and the majority of studies of GnRH signaling are performed in static culture using tonic treatment of GnRH. Therefore, a better understanding of the cellular decoding of the pulsatile GnRH signal is essential to elucidate the mechanisms that lead to GnRH pulse frequency dependent differential stimulation of synthesis and secretion of FSH and LH.

The function of GnRH neurons is controlled and modulated by a wide range of neuronal systems within the brain, which transmit feedback signals of sex steroids and mediate the response to stress, metabolic status and season [13–15]. Previous studies have recently provided a better understanding of how GnRH synthesis and secretion is controlled by modulatory neuronal systems in the brain. To a large extent, this is due to the recognition of the roles played by kisspeptin and gonadotropin inhibitory hormone (GnIH) [16]. A large number of studies have documented that metabolic status, season, and stress were able to modulate the function of GnRH neurons [16], thereby affecting reproduction. However, there is no evidence to indicate how GnRH neurons modulate the metabolic status, season, and stress, or how the GnRH signalling pathway interacts with other related pathways. Therefore, an improved understanding of the molecular mechanism of GnRH on metabolic status is required to develop more specifically therapeutic approaches for metabolic disease.

The availability of online databases and computer algorithms makes it possible to investigate the complex mechanisms of pathways. Recently, gene expression profile data associated with the gonadotropin subunit genes have been studied by many researchers. However, the detailed mechanism, associated with the gonadotropin response to GnRH, has not been studied further in a bioinformatics framework. In the present study, the signalling mechanisms of gonadotropin responses to GnRH were explored via a bioinformatics approach using the microarray data GSE63251signalling. Differentially expressed genes (DEGs) were firstly identified between cells with and without GnRH, followed by pathway enrichment analysis and construction of protein–protein interaction networks (PPI). Furthermore, the Gene Ontology (GO) function and Kyoto Encyclopedia of Genes and Genomes (KEGG) pathway enrichment analyses of the most remarkable module were performed using the Database for Annotation, Visualization and Integrated Discovery (DAVID) software. To our knowledge, this is the first report in which GnRH have been studied in the context of network biology. In addition, on the basis of networks, we proposed the hypothesis that GnRH was able to modulate the metabolic state, via the related membrane receptor, which was verified in the current study. The findings of this study may provide a systematic perspective to understand underlying mechanisms and a new insight of the GnRH role in gonadotropin subunit gene transcriptions and metabolic balance.

## Methods

### Affymetrix microarray data

The microarray data of GSE63251, contributed by Lawson et al. [17], were downloaded from the Gene Expression Omnibus (GEO) (http://www.ncbi.nlm.nih.gov/geo/) database at the National Center for Biotechnology Information (NCBI), which is currently the largest fully public gene expression resource [18]. The microarray data GSE63251 contains 20 samples, including two samples of un-stimulated, four samples of tonic GnRH, and fourteen different GnRH dose and pulse frequencies. Only six only samples of these were analysed in the present study, including two samples of un-stimulated, two samples of perfusion tonic 100 nM GnRH and two samples of perfusion 8 pulses 100 nM GnRH based on the platform of GPL81 [MG_U74Av2] Affymetrix Murine Genome U74A Version 2 Array.

### Data preprocessing and differential expression analysis

Background correction and quartile data normalization was performed on the original array data [19]. The DEGs (DEG1) of GnRH pulse and the DEGs (DEG2) of GnRH tonic were respectively analysed with respect to the control group. Multiple testing was corrected using the Benjamini–Hochberg [20] procedure to obtain the adjusted *P*-value. Fold changes (FCs) in the expression of individual genes were calculated and DEGs with *P* < 0.05 and |log FC| >1 were considered to be significant. DEG1 and DEG2 were then combined and the pooled dataset was referred to as the overlapping DEGs.

### Functional and pathway enrichment analysis

The Kyoto Encyclopedia of Genes and Genomes (KEGG) [21] knowledge database is a collection of online databases dealing with genomes, enzymatic pathways, and biological chemicals. The Database for Annotation, Visualization and Integrated Discovery (DAVID) [22], has been developed as a comprehensive set of functional annotation tools for relating functional terms with gene lists using a clustering algorithm. The Gene Ontology database provides functional unification of large-scale genomic or transcriptomic data, which mainly includes three categories, including biological processes (BP), molecular functions (MF), and cellular components (CC). KEGG pathways of the overlapping DEGs were analysed with a criterion of *P* < 0.05 using DAVID 6.7.

### PPI network construction and module selection

The Search Tool for the Retrieval of Interacting Genes (STRING, version 9.1, http://www.string-db.org/) [23] is a web resource and biological database that includes comprehensively predicted and known interaction information. In our study, the overlapping DEGs with a confidence score of >0.4 were selected to construct the PPI network using Cytoscape software (version 3.0; http://cytoscape.org/) [24]. Given that most of the networks were scale-free, the hub genes were then selected with a connectivity degree of >10. Then, a biological network was created between the GnRH signalling pathway and other related pathways by hub genes. In addition, based on the degree of connectivity, the enriched functional modules of the PPI network were disclosed using the ClusterONE Cytoscape plug-in [25, 26]. The significance threshold was *P* < 1 × 10^−4^. Furthermore, the GO and KEGG pathway enrichment analyses of the DEGs in the significant modules were performed using DAVID to analyse the gene function at the molecular level.

### Cell culture

LβT2 cells, a gift from Dr. Pamela Mellon (University of California, San Diego) were maintained in high-glucose DMEM (Mediatech, Inc), supplementedwith 10%foetal bovine serum (FBS) (Gibico), 100 U of penicillin/mL, and 100 μg of streptomycin sulphate/mL (Thermo Fisher Scientific, Inc) in 5% CO2 humidified air at 37 °C [27].

### Verifying the mechanisms of metabolic pathways in response to GnRH in vitro

Mouse GnRH-10 was synthesized by Shanghai Apeptide Co Ltd. The interferencethe vectors for LEPR and INSR were constructed. The sequences that express short hairpin RNA (shRNA) targeted to gene mRNA sequences were cloned in a p-SUPER vector (the Sigma-Aldrich) (http://www.sigmaaldrich.com/china-mainland.html) according to manufacturer’s instructions.

LβT-2 cells were cultured at a in a concentration of 1 × 10^5^ viable cells/ml culture medium, in 12-well plates at 37 °C and 5% CO_2_. After 24 h, the medium was replaced with RPMI only, and then 1.5 μg of empty pSUPER(with a negative control), LEPR-shRNA or INSR-shRNA vectors were transfected using Lipofectamine 2000. Approximately 8 h after transfection, the cells were serum starved for 8 h and later stimulated with GnRH for 24 h, and then total RNA was extracted using TRIzol reagent.

### Reverse transcription quantitative real-time PCR (RT-qPCR)

RNA was extracted using TRIzol reagent (TaKaRa), and 1 μg of total RNA was reverse transcribed into first-strand cDNA with a mix of oligo-dT and random primers using the Quantitect Reverse Transcription Kit (TaKaRa). RT-qPCR was performed on an Applied Biosystems 7500 Real-Time PCR System using SYBR green. Standard curves, generated from at least three dilution series of an abundant sample, were used for the relative quantification of related genes and β-actin (the same sample was used to generate standard curves for these genes). The expression levels of genes were normalized by dividing by β-actin mRNA expression levels (primers are shown in Table 1). Dissociation curve analysis was also performed for each gene at the end of PCR. Each amplicon generated a single peak. PCR cycling conditions were as follows: initial denaturation and enzyme activation at 95 °C for 30 s, followed by 40 cycles of denaturation at 95 °C for 5 s, and annealing at 60 °C for 30 s.

**Table 1:** The sequences of primers for quantitative fluorescent RT-PCR

Genes	Primers	Length of target fragment, bp
β-Actin	F: 5′- ACTCCTATGTGGGTGACGAGG-3′	137
	R: 5′-CACACGCAGCTCATTGTAGAAG-3′	
NPY	F:5′- CCAGACAGAGATATGGCAAG-3′	110
	R:5′- CATGGAAGGGTCTTCAAGCC-3′	
PTPN11	F:5′- TACGGGGTCATGCGTGTTAG-3′	125
	R:5′- GAAAGTGGTACTGCCAGACG-3′	
GYS1	F:5′- TGGTGGGACCATACACGGA-3′	113
	R:5′- TCAGCCAACGCCCAAAATAC-3′	
PKLR	F:5′- GCCGCATCTACATTGACGAC-3′	127
	R:5′- GCATTTGGCAAGTTCACACC-3′	
F, Forward; R, reverse.		

### Statistical analysis

All statistical analysis was performed using Graphpad Prism software. Data were analysed using unpaired Student’s *t*-test or one-way ANOVA and Tukey’s post-hoc test. Data are presented as means ± SEM of at least three independent experiments and *P* < 0.05 was considered statistically significant.

## Results

### Identification of DEGs

**Fig. 1:**
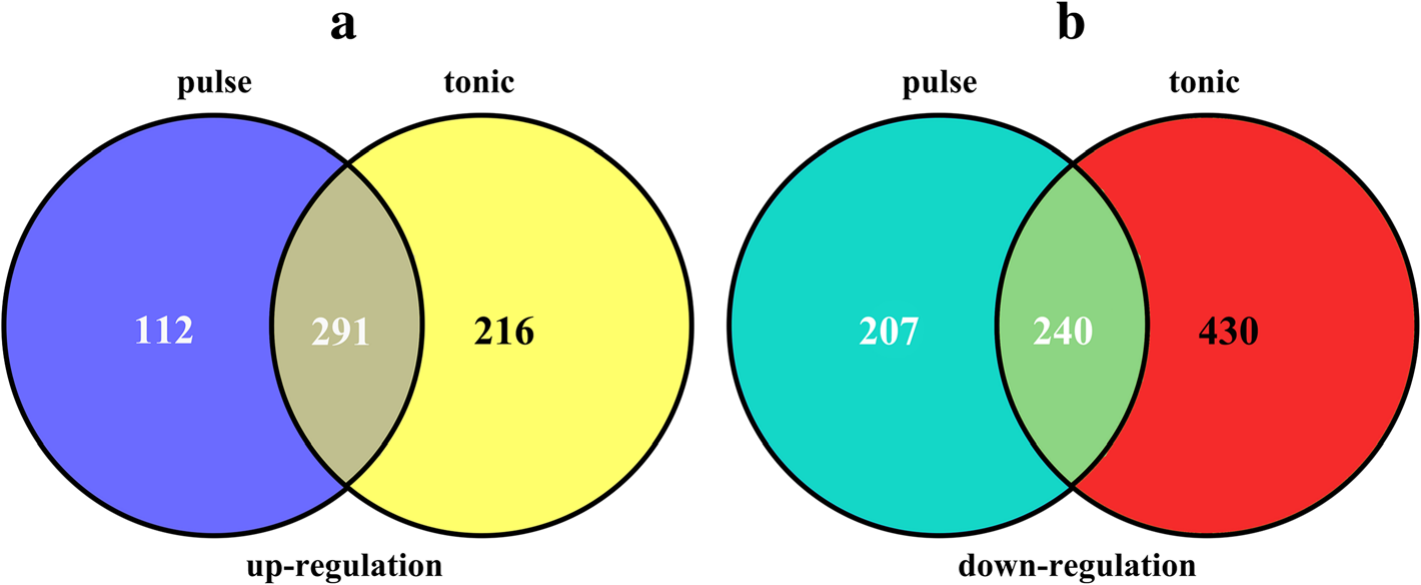
Schematic Venn diagram of differentially expressed genes. Schematic Venn diagram of differentially expressed genes between the GnRH group and control group. **a** Up-regulated DEGs: *blue* and *yellow* represent DEGs in the pulse GnRH and tonic GnRH groups respectively, compared with controls. **b** Down-regulated DEGs: *green* and *red* represent DEGs in the pulse GnRH and tonic GnRH groups, respectively, compared with controls

The neuropeptide hormone GnRH is a hypothalamic peptide essential for normal mammalian reproductive function. Gonadotropin synthesis and release is dependent upon pulsatile stimulation by the hypothalamic neuropeptide GnRH. However, the role of GnRH pulse frequency was not examined in depth. This study investigated the gene profiles that responded to GnRH. Using bioinformatics approaches, a total of 1177 DEGs between perfusion tonic 100 nM GnRH and control group with |logFC| > 1 and *P* < 0.05 were identified, including 507 up-regulated genes such as jun proto-oncogene (JUN), and activating transcription factor 4 (ATF 4) and 670 down-regulated DEGs, such as insulin receptor substrate 1 (IRS 1). In addition, a total of 403 up-regulated (such as activating transcription factor 3) and 447 down-regulated (such as inositol 1,4,5-trisphosphate receptor 2) DEGs were identified between the GnRH pulses group and control group. The schematic Venn diagram of differentially expressed genes is shown in Fig. 1.

### Pathway enrichment analysis

To gain further understanding of the potential functional roles of the DEGs in GnRH treatment, this study performed a KEGG pathways analysis and set *P* < 0.05 as the cut-off value. The results are shown in Table 2. According to the results, up-regulated genes were primarily enriched in pathways associated with MAPK signalling pathway, p53 signalling pathway, metabolic pathways, adipocytokine signalling pathway and GnRH signalling pathway (Table 2). Down-regulated genes were primarily enriched in pathways associated with oocyte meiosis, progesterone-mediated oocyte maturation and type II diabetes mellitus (Table 2). However, the mechanisms of how gonadotropes are affected by GnRH pulse frequency and amplitude is not understood. Therefore, this study was concerned with the differences in gene expression in GnRH pulse and GnRH tonic, and mainly focused on the changes that occurred inGnRH pulse, as these were likely occurred in vivo. Then KEGG pathways analysis showed that DEGs unique to the GnRH pulse group were DEGs mainly enriched in dilated cardiomyopathy, hypertrophic cardiomyopathy, Alzheimer’s disease and porphyrin and chlorophyll metabolism, which were associate with disease (Table 3).

**Table 2:** The enriched KEGG pathway jof DEGs

Up-Regulated					
ID	Term	Counts		*P*-value	
		Pulse	Constant	Pulse	Constant
mmu04010	MAPK signalling pathway	25	34	3.03E-05	6.49E-09
mmu05412	Arrhythmogenic right ventricular cardiomyopathy (ARVC)	11	9	3.60E-04	0.011371
mmu04520	Adherens junction	11	11	4.01E-04	1.00E-03
mmu04115	p53 signalling pathway	10	10	8.34E-04	1.90E-03
mmu04510	Focal adhesion	17	15	0.002282	0.032455
mmu05200	Pathways in cancer	23	21	0.003327	0.04242
mmu01100	Metabolic pathways	83	111	0.015105	0.008450594
mmu04060	Cytokine-cytokine receptor interaction	19	11	0.033202668	0.048153254
mmu04920	Adipocytokine signalling pathway	7	8	0.034984	0.019396
mmu05020	Prion diseases	5	6	0.037602	0.013348
mmu05416	Viral myocarditis	8	9	0.045733	0.038737
mmu04912	GnRH signalling pathway	11	11	0.046921	0.045341
mmu04012	ErbB signalling pathway	7	9	0.048712	0.025918
Down-Regulated					
mmu00280	Valine, leucine and isoleucine degradation	8	0.004814562	6	0.049249252
mmu04114	Oocyte meiosis	13	0.006667	12	0.03641
mmu04914	Progesterone-mediated oocyte maturation	10	0.016259	10	0.031867499
mmu04930	Type II diabetes mellitus	7	0.024595	8	0.012683147

Notes: Count: the number of DEGs

Abbreviations: *DEGs* differentially expressed genes

**Table 3:** The enriched KEGG pathway of DEGs which exist in pulse GnRH group while not exist in tonic GnRH group

ID	Term	Count	*P*-value
mmu05410	Hypertrophic cardiomyopathy (HCM)	10	0.003372782
mmu05414	Dilated cardiomyopathy	10	0.00618965
mmu04670	Leukocyte transendothelial migration	9	0.049331716
mmu04640	Hematopoietic cell lineage	11	0.005141872
mmu05010	Alzheimer’s disease	15	0.042214081
mmu00860	Porphyrin and chlorophyll metabolism	5	0.047944857
mmu04020	Calcium signalling pathway	15	0.048934483

Notes: Count: the number of DEGs

Abbreviations: *DEGs* differentially expressed genes

### PPI network construction and hub gene identification

**Fig. 2:**
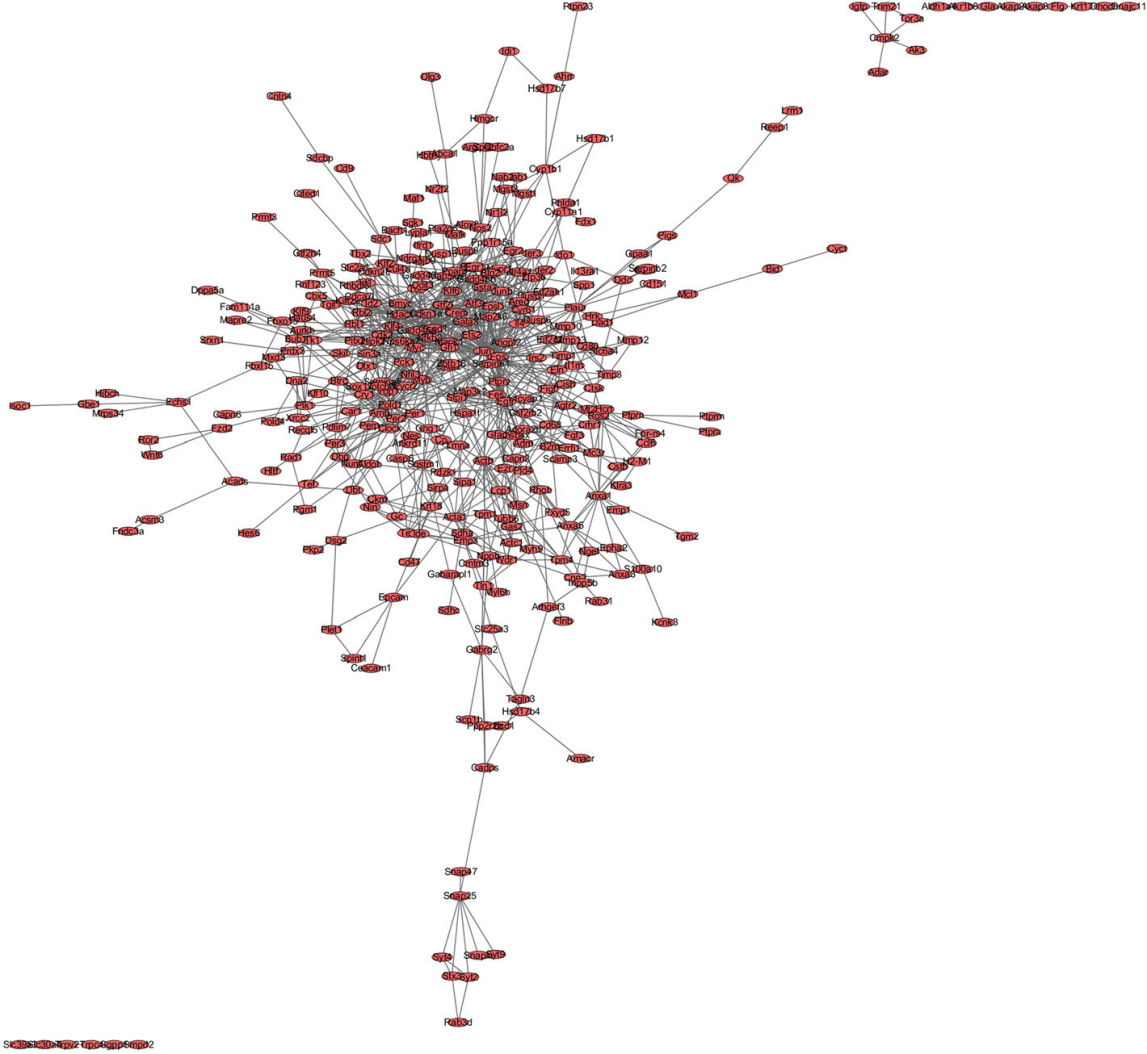
Protein–protein interaction networks of differentially expressed genes (DEGs). The size of each node is proportional to the degree of nodes. DEG, differentially expressed gene

**Fig. 3:**
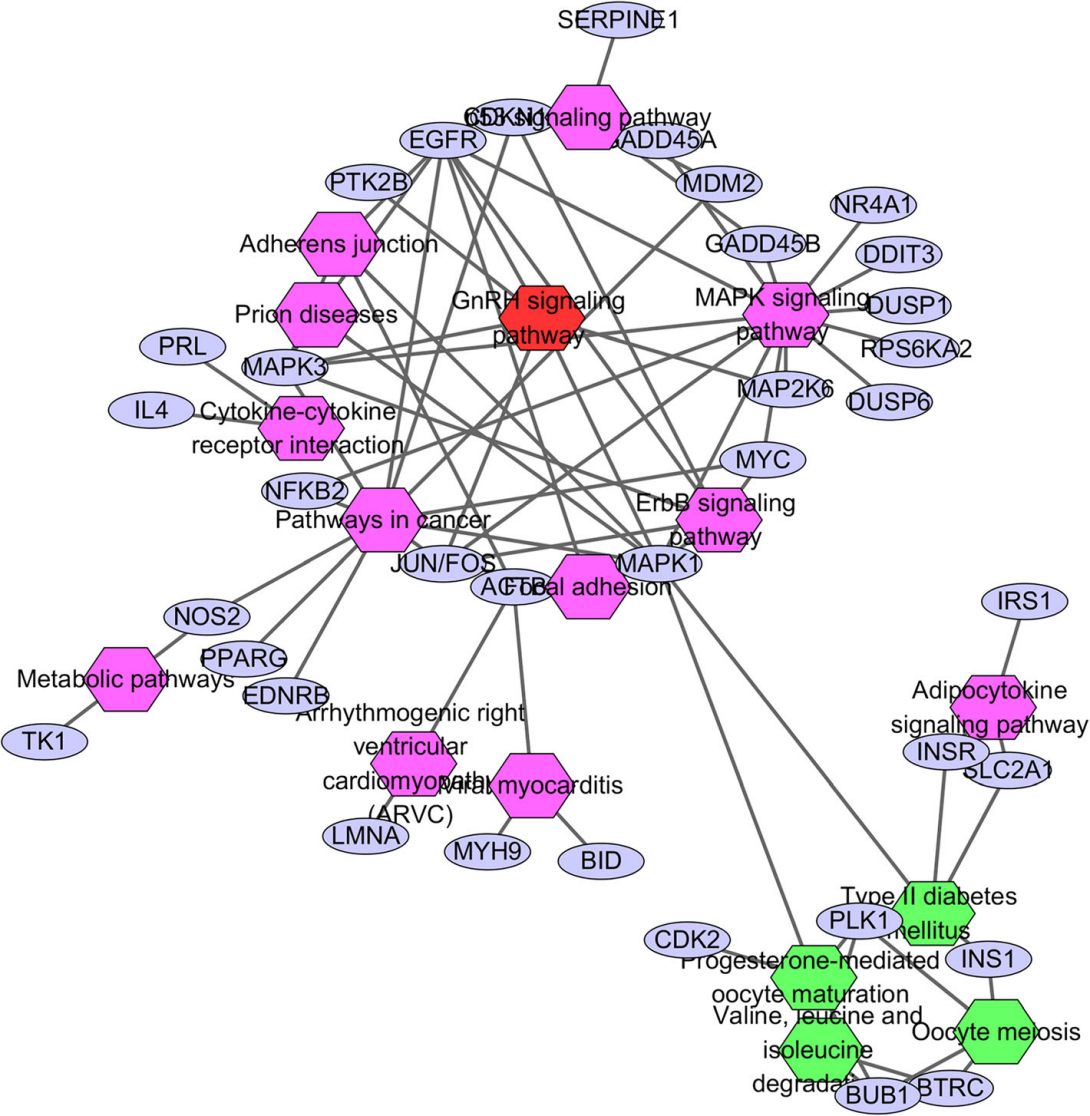
Nodes linking the enriched KEGG pathway by hub genes. *Hexagon* represents each pathway. *Round circle* indicates hub genes. *Pink hexagons* represent up-regulated signalling pathway, and *green hexagons* represent down-regulated signalling pathway

Considering that proteins rarely function alone, it is necessary to study the protein–protein interactions. Therefore, protein-protein interactions were constructed in the present study, which helped to understand functions of proteins at the molecule level and explore cell regulatory mechanisms. Based on the STRING database, the PPI network of overlapping DEGs contained 334 nodes and 989 edges network were gained with the combined score > 0.4 using Cytoscape (Fig. 2). Proteins with a very high degree of connectivity are commonly called “hubs”, and they may play a central regulatory role in the physiological process. Then, the hub genes or proteins in the networks with a connectivity degree of >10 were identified (Table 4, top10). A total of 53 hub genes were selected from the PPI network, which included FBJ osteosarcoma oncogene (FOS), jun proto-oncogene (JUN), mitogen-activated protein kinase 1 (MAPK 1), epidermal growth factor receptor (EGFR), and activating transcription factor 3 (ATF 3). In addition, the result of pathway enrichment showed that common DEGs were mainly enriched in seventeen pathways, but how these signalling pathways interplay with the GnRH signalling pathway are still ambiguous. Since hub genes play a crucial role in many process, we proposed the hypothesis that these signalling pathways interplayed with GnRH signalling pathway mainly through hub genes. Based on this, we created a biological network to analyse the interaction between the signalling pathways through hub genes using Cytoscape tool (Fig. 3). In fact, the biological network showed that these signalling pathways were directly or indirectly linked to each other through hub genes; for example, GnRH signalling pathways may regulate INS1 release and type II diabetes mellitus via the MKPA signalling pathway.

**Table 4:** The hub proteins in protein–protein interaction network (top 10)

ID	Degree	ID	Degree
Fos	59	Mapk1	34
Jun	56	Mapk3	34
Myc	40	Atf3	29
Egfr	37	Egr1	27
Cdkn1a	36	Junb	26

### Functional module analysis

**Fig. 4:**
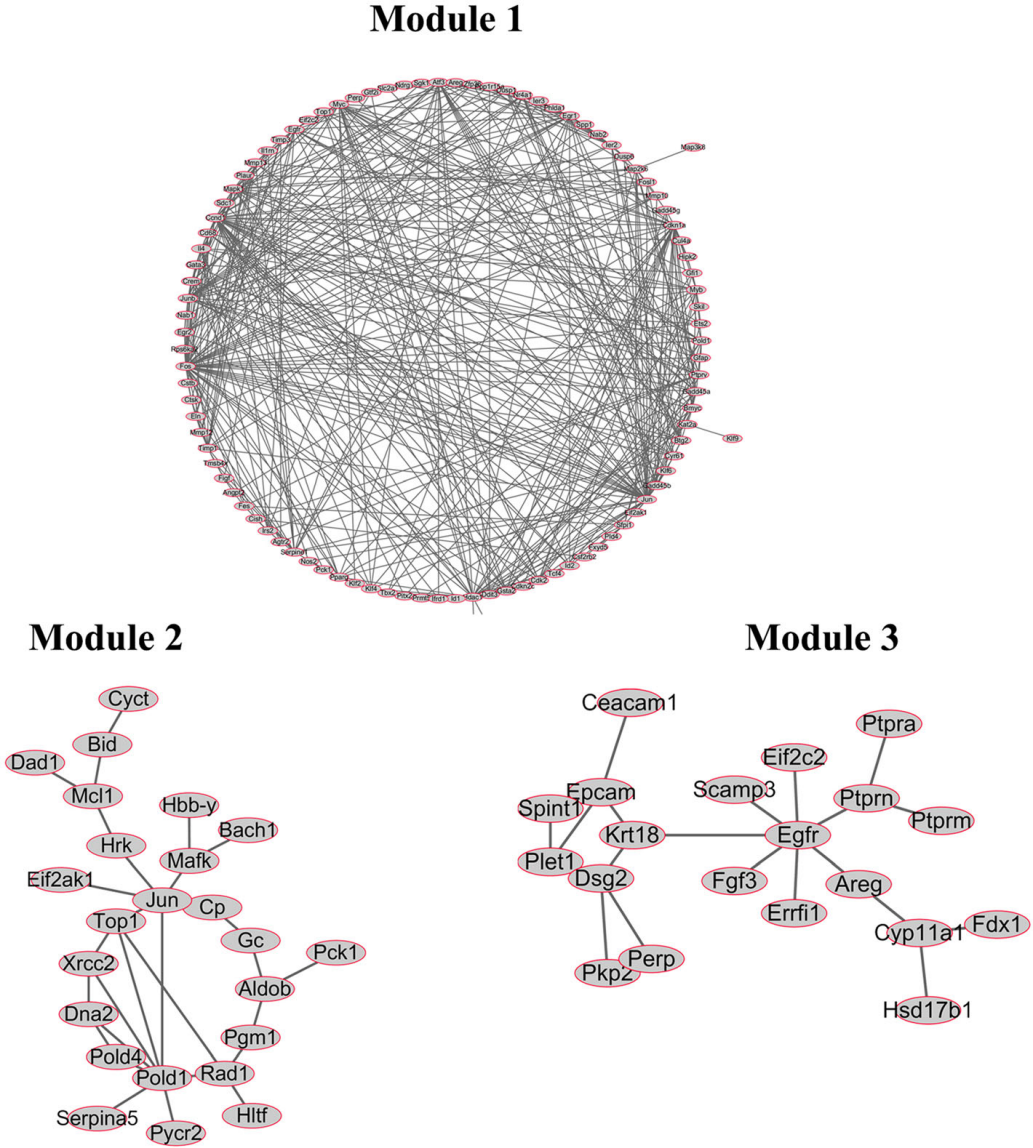
The significant modules in protein-protein interaction network with ClusterONE (*p*<10E-5). The node stands for the protein (gene), The edges/lines indicate interactions between these genes

**Fig. 5:**
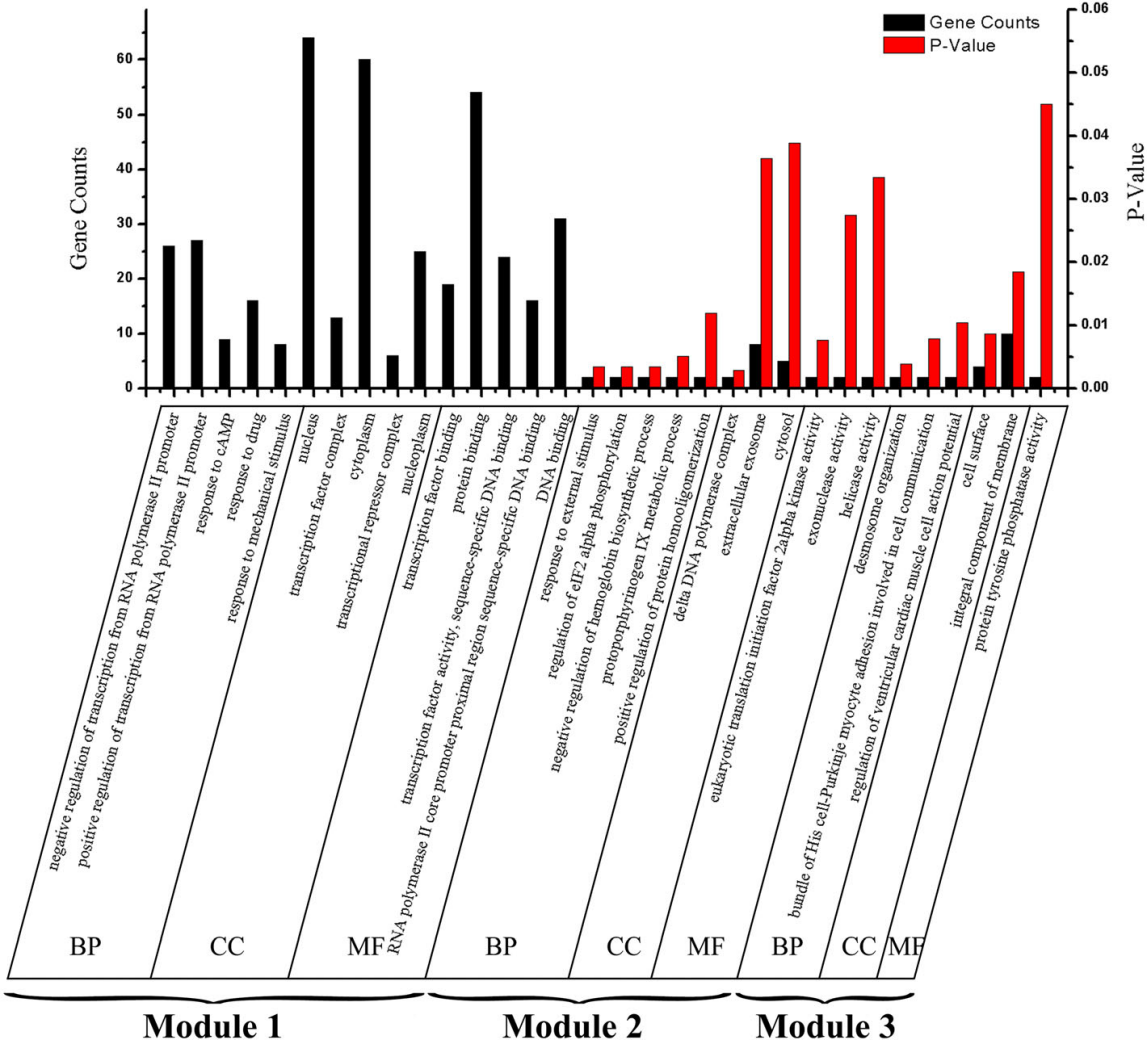
The Gene Ontology (GO) functional of the significant module. The *left* ordinate of histogram represents the gene counts, and *right* ordinate of histogram represents the *P*-value. BP, biological process; CC, cellular component; MF, molecule function

In order to explore the functions of the networks modules, we implemented cluster analysis. As shown in Fig. 4, a total of three significant modules with *P* < 1 × 10^−4^were identified using the ClusterONE plug-in of Cytoscape. Module 1 had 99 nodes and 460 edges, module 2 had 24 nodes and 29 edges and module 3 had 20 nodes and 21 edges. KEGG enrichment in the sub-network is presented in Table 5. The FoxO signalling pathway, MAPK signalling pathway, insulin resistance and GnRH signalling pathway were the predominant pathways enriched by the DEGs of module 1. The pathways enriched in module 2 were the glycolysis/gluconeogenesis, biosynthesis of antibiotics and metabolic pathways. Module 3 was mainly enriched in steroid hormone biosynthesis and ovarian steroidogenesis pathways. GO enrichment analysis was also performed and is presented in Fig. 5. The DEGs in module 1 were significantly enriched in biological processes, such as the regulation of transcription from RNA polymerase II promoter, response to drugs. signallingDEGs in module 2 were mainly enriched in GO terms associated with response to external stimuli, for example, regulation of eIF2 alpha phosphorylation and negative regulation of haemoglobin biosynthetic processes, while DEGs in module 3 were mainly enriched in cell surface and protein tyrosine phosphatase activity.

**Table 5:** Enriched pathways of DEGs in sub-network

Category	Term	Count	*P*-value
Module 1			
KEGG_PATHWAY	FoxO signalling pathway	12	2.21E-08
KEGG_PATHWAY	MAPK signalling pathway	15	3.56E-08
KEGG_PATHWAY	GnRH signalling pathway	6	0.04375722
KEGG_PATHWAY	Estrogen signalling pathway	5	0.046988099
KEGG_PATHWAY	Insulin resistance	5	0.045070173
Module 2			
KEGG_PATHWAY	Homologous recombination	3	0.001471
KEGG_PATHWAY	DNA replication	3	0.00239
KEGG_PATHWAY	Glycolysis / Gluconeogenesis	3	0.00723
KEGG_PATHWAY	Biosynthesis of antibiotics	4	0.007747
KEGG_PATHWAY	Metabolic pathways	7	0.023793
Module 3			
KEGG_PATHWAY	Steroid hormone biosynthesis	2	0.069411
KEGG_PATHWAY	Ovarian steroidogenesis	2	0.073434

Count, numbers of DEGs; *GO* gen ontology, *KEGG* Kyoto Encyclopedia of Genes and Genomes

### Verifying the mechanisms of metabolic pathways in response to GnRH in vitro

**Fig. 6:**
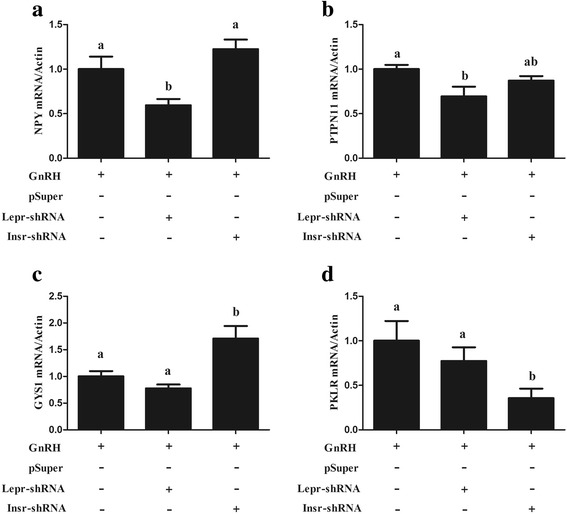
Effects of GnRH on metabolic related genes expression by knockdown LEPR or INSR. The LβT2 cells transiently transfected with shRNAs vector targeting LEPR, INSR or empty vector after GnRH treatment. Then total RNA was extracted for qRT-PCR analysis of the genes expression. A, NPY related expression; B, PTPN11 related expression; C, GYS1 related expression; D, PKLR related expression

To date, many studies have confirmed that metabolic status, season, and stress were able to modulate the function of GnRH neurons [13–15]. However, there was no evidence that indicated how GnRH neurons modulate the metabolic status, season, and stress. Therefore, this study investigated that the regulatory function of GnRH on metabolic status. It is known thatleptin, a peptide hormone secreted by adipocytes, is encoded by the “fat gene” [28] which can increase body energy expenditure along with decreased energy intake by suppressing appetite [29], thus playing a crucial role in metabolism and body growth mediation. Based on this, we investigated whether GnRH modulates the energy expenditure and energy intake via LEPR, by knockdown of LEPR expression. The results showed that the GnRH-induced *NPY* (Fig. 6a) and *PTPN11* (Fig. 6b) expression was significantly suppressed when the expression of *LEPR* was interfered, genes which are responsible for energy intake and reproduction, respectively. In addition, the role of GnRH on the insulin signalling pathway was also detected. Interfering with LEPR facilitated the GnRH-suppressed *GYS1* expression (Fig. 6c), which promoted glycogenesis. Conversely, interfering with LEPR significantly repressed GnRH-induced *PKLR* expression (Fig. 6d), which promoted glycolysis.

## Discussion

GnRH plays a central role in the control of normal reproductive function by regulating the synthesis and release of the gonadotropins LH and FSH [30–32]. The knownlege of signalling mechanisms involved in gonadotropin synthesis have been expanding. However, the mechanisms underlying gonadotropin regulation by GnRH remained to be fully elucidated. Bioinformatics tools have accelerated the progression of biomedical research, with high-throughput screening being especially useful. Such tools not only reduce the need for time-consuming and labour-intensive bench-work, but also provide critical directions and insights for advanced studies [33]. In this study, we first systematically explored the mechanisms of gonadotropin responding to GnRH by bioinformatics. While standard pathway analysis investigates each pathway individually, biological processes are not independent but rather interact with and influence each other. Therefore, it is necessary to investigate the links between them as well as their shared regulatory mechanisms. In our study, the mechanism was analysed by bioinformatics, including DEG screening, PPI network construction, and module analysis of the PPI network. Based on these results, a workflow was described for further exploring the interplay between pathways involved in the gonadotropin regulatory mechanisms, through the construction of a biological network and extending the GnRH signalling pathway with additional knowledge involved in other physiological processes.

In the present study, the results of the GO and KEGG pathway enrichment analyses revealed the primary biological processes in which the DEGs were involved, including focal adhesion, type II diabetes mellitus, glycogen biosynthesis and metabolism, progesterone-mediated oocyte maturation. Furthermore, other pathways associated with GnRH regulation were activated simultaneously followed by treatment with GnRH, such as MAPK signalling pathway, cytokine-cytokine receptor interaction, and most importantly, the GnRH signalling pathway [34]. Based on the results, it was revealed that the GnRH signalling pathway interplayed with other signalling pathways to co-regulate the reproductive function and other biological process.

Metabolic dysfunctions are often linked to reproductive abnormalities [35]. Obesity and conditions with hyperinsulinemia such as type 2 diabetes mellitus, metabolic syndrome, and polycystic ovary syndrome (PCOS) are often accompanied by infertility in females [36]. Insulin, like LH, is a gonadotropin, which increases steroidogenesis and altered follicular maturation in animal models [37]. In addition to insulin, leptin also served as a critical factor in the process of fat cell metabolism, which can convey information about nutritional state to the reproductive axis [35]. In a normal metabolic state, leptin increases GnRH activity/secretion (indirectly via afferent forebrain interneurons) [38] and enhances reproductive function in females [39]. In cases of metabolic dysfunction, like obesity, leptin resistance can occur [40] eliminating a positive signal to the reproductive axis. However, there was previously no evidence to indicate how GnRH neurons modulate the metabolic status. Based on this, we proposed the hypothesis that GnRH may modulate the metabolic status through regulating expression of the related genes, such as the membrane receptor LEPR and INSR. In fact, the current results showed that interfering with *LEPR* significantly suppressed GnRH-induced NPY and PTPN11 expression, which are responsible for energy intake and reproduction, respectively. Similarly, Interfering with LEPR facilitated the GnRH-suppressed GYS1 expression, which promoted the glycogenesis. Conversely, interfering with LEPR significant repressed GnRH-induced PKLR expression, which promoted glycolysis. These results provide a better understanding of the regulation of GnRH on energy intake and expenditure at the molecular level, which constitute useful insights into the relationship between reproduction and metabolic status.

Previous studies have demonstrated that different frequencies of pulsatile GnRH result in altered secretion patterns of FSH and LH. Increasing frequencies results in preferential secretion of LH, whereas decreasing frequencies leads to greater FSH secretion. Because FSH and LH synthesis and secretion are both modulated by GnRH through the same G protein-coupled GnRH receptor, one possible mechanism is that distinct signalling pathways are activated, depending on GnRH pulse frequency, leading to the differential regulation of FSH and LH production [41, 42]. However, it remains unknown how gonadotropes decode the pulsatile GnRH signal to preferentially synthesize FSH or LH. In the present study, the difference between GnRH pulse and GnRH tone were also analysed. As expected, GO analysis and KEGG pathway enrichment analysis revealed that GnRH pulse treatment activated the unique pathways. These pathways are involved in hypertrophic cardiomyopathy, dilated cardiomyopathy, alzheimer’s disease, as well as the calcium signalling pathway. These results further indicated that abnormal GnRH pulse and amplitude may cause disease, which may provide an improved understanding of the GnRH pathway and a new insight for disease diagnosis and treatment.

However, there are some limitations in our study. First, the small amounts of data used in the current study were downloaded from the GEO database, not generated by us. Because GEO is a huge data repository, a meta-analysis of the relevant molecular mechanism of GnRH may be performed in future studies. Second, the results were web-based and were not all verified by biological experiments. Thus, further experimental studies based on our findings are still needed.

## Conclusions

In summary, with this study, for the first time, we characterized GnRH to prove an insight into their role in gonadotropin regulation and metabolic balance using PPI networks and GO and KEGG enrichment analysis, which might provide comprehensive bioinformatics analysis of the mechanisms of GnRH. These results indicated that the GnRH signalling pathway interplayed with other signalling pathways to co-regulate the reproductive function and other biological process. In addition, the difference between GnRH pulse and GnRH tone revealed that abnormal GnRH pulse and amplitude may cause disease, which may provide an improved understanding of the GnRH pathway and a new insight for disease diagnosis and treatment. However, further experimentation and additional studies are needed to validate these results.

## Abbreviations

BP: Biological process
CC: Cellular component GnRH: gonadotropin­releasing hormone
DAVID: Database for annotation, visualization and integrated discovery
FSH: Follicle-Stimulating hormone
GEO: Gene expression omnibus
GO: Gene ontology
HPG: Hypothalamic-pituitary-gonadal
KEGG: Kyoto encyclopedia of genes and genomes
LH: Luteinizing hormone
MF: Molecular function
PPI: protein-protein interaction

## References

[1] Thackray VG, Mellon PL, Coss D (2010). Hormones in synergy: regulation of the pituitary Gonadotropin genes. Mol Cell Endocrinol.

[2] Millar RP, Newton CL (2013). Current and future applications of GnRH, kisspeptin and neurokinin B analogues. Nat Rev Endocrinol.

[3] Stanislaus D, Pinter JH, Janovick JA, Conn PM (1998). Mechanisms mediating multiple physiological responses to gonadotropin-releasing hormone. Mol Cell Endocrinol.

[4] Alarid ET, Windle JJ, Whyte DB, Mellon PL (1996). Immortalization of pituitary cells at discrete stages of development by directed oncogenesis in transgenic mice. Development.

[5] Christian CA, Moenter SM (2010). The neurobiology of preovulatory and estradiol-induced gonadotropin-releasing hormone surges. Endocr Rev.

[6] Herbison AE (2008). Estrogen positive feedback to gonadotropin-releasing hormone (GnRH) neurons in the rodent: the case for the rostral periventricular area of the third ventricle (RP3V). Brain Res Rev.

[7] Petersen SL, Ottem EN, Carpenter CD (2004). Direct and indirect regulation of gonadotropin-releasing hormone neurons by estradiol. Biol Reprod.

[8] Millar RP, Lu ZL, Pawson AJ, Flanagan CA, Morgan K, Maudsley SR (2004). Gonadotropin-releasing hormone receptors. Endocr Rev.

[9] Savoy-Moore RT, Swartz KH (1987). Several GnRH stimulation frequencies differentially release FSH and LH from isolated, Perfused rat anterior pituitary cells. Adv Exp Med Biol.

[10] Thompson IR, Ciccone NA, Zhou Q, Xu S, Khogeer A, Carroll RS (2016). GnRH pulse frequency control of *Fshb* Gene expression is mediated via ERK1/2 regulation of ICER. Mol Endocrinol.

[11] Burger LL, Dalkin AC, Aylor KW, Haisenleder DJ, Marshall JC (2002). GnRH pulse frequency modulation of Gonadotropin subunit Gene transcription in normal Gonadotropes—assessment by primary transcript assay provides evidence for roles of GnRH and Follistatin. Endocrinology.

[12] Dalkin AC, Haisenleder DJ, Ortolano GA, Ellis TR, Marshall JC. The frequency of gonadotropin-releasing-hormone stimulation differentially regulates gonadotropin subunit messenger ribonucleic acid expression. 1989;125:917–24. 10.1210/endo-125-2-917 2502379

[13] Clarke IJ, Caraty A (2013). Kisspeptin and seasonality of reproduction. Adv Exp Med Biol.

[14] Clarke IJ, Tilbrook AJ. Gonadotropin, neural and hormonal control. Encycl Neurosci. 2009:959–65.

[15] Clarke IJ (2014). Interface between metabolic balance and reproduction in ruminants: focus on the hypothalamus and pituitary. Horm Behav.

[16] Clarke IJ, Arbabi L (2016). New concepts of the central control of reproduction, integrating influence of stress, metabolic state, and season. Domest Anim Endocrinol.

[17] Lawson MA, Tsutsumi R, Zhang H, Talukdar I, Butler BK, Santos SJ (2007). Pulse sensitivity of the luteinizing hormone β promoter is determined by a negative feedback loop involving early growth response-1 and Ngfi-a binding protein 1 and 2. Mol Endocrinol.

[18] Barrett T (2011). NCBI GEO: archive for functional genomics data sets—update. Nucleic Acids Res.

[19] Hulsegge I, Kommadath A, Smits MA (2009). Globaltest and GOEAST: two different approaches for Gene ontology analysis. BMC Proc.

[20] Wang Y, Huang L, Wu S, Jia Y, Yang Y, Luo L (2014). Bioinformatics analyses of the role of vascular endothelial growth factor in patients with non-small cell lung cancer. PLoS One.

[21] Du J, Yuan Z, Ma Z, Song J, Xie X, Chen Y (2014). KEGG-PATH: Kyoto encyclopedia of genes and genomes-based pathway analysis using a path analysis model. Mol BioSyst.

[22] Huang DW, Sherman BT, Lempicki RA (2009). Systematic and integrative analysis of large gene lists using DAVID bioinformatics resources. Nat Protoc.

[23] Mering CV, Huynen M, Jaeggi D, Schmidt S, Bork P, Snel B (2003). STRING: a database of predicted functional associations between proteins. Nucleic Acids Res.

[24] Saito R, Smoot ME, Ono K, Ruscheinski J, Wang PL, Lotia S (2012). A travel guide to Cytoscape plugins. Nat Methods.

[25] Deng L, Xiong P, Luo Y, Bu X, Qian S, Zhong W. Bioinformatics analysis of the molecular mechanism of diffuse intrinsic pontine glioma. Oncol Lett. 2016; [cited 2017 Feb 22]; Available from: http://www.spandidos-publications.com/10.3892/ol.2016.5024 10.3892/ol.2016.5024 PMC5038193 27698822

[26] Maraziotis IA, Konstantina D, Anastasios B (2008). An in silico method for detecting overlapping functional modules from composite biological networks. BMC Syst Biol.

[27] Son YL, Ubuka T, Millar RP, Kanasaki H, Tsutsui K (2012). Gonadotropin-inhibitory hormone inhibits GnRH-induced Gonadotropin subunit Gene transcriptions by inhibiting AC/cAMP/PKA-dependent ERK pathway in LβT2 cells. Endocrinology.

[28] Mann DR, Plant TM (2002). Leptin and pubertal development. Semin Reprod Med.

[29] Luo Q, Li W, Li M, Zhang X, Zhang H (2016). Leptin/leptinR-kisspeptin/kiss1r-GnRH pathway reacting to regulate puberty onset during negative energy balance. Life Sci.

[30] Kaiser UB, Conn PM, Chin WW (1997). Studies of gonadotropin-releasing hormone (GnRH) action using GnRH receptor-expressing pituitary cell lines. Endocr Rev.

[31] Stuart P, Bliss AMN (2010). GnRH signalling, the gonadotrope and endocrine control of fertility. Front Neuroendocrinol.

[32] Ioan A, Ciuleanu T, Guttman T, Ghilezan N (2000). Development of GnRH antagonists for prostate cancer: new approaches to treatment. Oncologist.

[33] Kutmon M, Evelo CT, Coort SL (2014). A network biology workflow to study transcriptomics data of the diabetic liver. BMC Genomics.

[34] Ye R-S, Xi Q-Y, Qi Q, Cheng X, Chen T, Li H (2013). Differentially expressed miRNAs after GnRH treatment and their potential roles in FSH regulation in porcine anterior pituitary cell. Kim YK, editor. PLoS One.

[35] Klenke U, Taylor-Burds C, Wray S (2014). Metabolic influences on reproduction: Adiponectin attenuates GnRH neuronal activity in female mice. Endocrinology.

[36] Michalakis K, Mintziori G, Kaprara A, Tarlatzis BC, Goulis DG (2013). The complex interaction between obesity, metabolic syndrome and reproductive axis: a narrative review. Metab Clin Exp.

[37] Wu S, Divall S, Nwaopara A, Radovick S, Wondisford F, Ko C (2014). Obesity-induced infertility and hyperandrogenism are corrected by deletion of the insulin receptor in the ovarian theca cell. Diabetes.

[38] Quennell JH, Mulligan AC, Tups A, Liu XH, Phipps SJ, Kemp CJ (2009). Leptin indirectly regulates gonadotropin-releasing hormone neuronal function. Endocrinology.

[39] Hausman GJ, Barb CR, Lents CA (2012). Leptin and reproductive function. Biochimie.

[40] Ahima RS, Qi Y, Singhal NS (2006). Adipokines that link obesity and diabetes to the hypothalamus. Prog Brain Res.

[41] Liu F, Usui I, Evans LG, Austin DA, Mellon PL, Olefsky JM (2002). Involvement of both G(q/11) and G(s) proteins in gonadotropin-releasing hormone receptor-mediated signalling in L beta T2 cells. J Biol Chem.

[42] Krsmanovic LZ, Mores N, Navarro CE, Arora KK, Catt KJ (2003). An agonist-induced switch in G protein coupling of the gonadotropin-releasing hormone receptor regulates pulsatile neuropeptide secretion. Proc Natl Acad Sci U S A.

